# Understanding the Relationship between Illness Perceptions and Health Behaviour among Women with Polycystic Ovary Syndrome

**DOI:** 10.3390/ijerph20115998

**Published:** 2023-05-30

**Authors:** Brittany Fossey, Kirsten J. McCaffery, Erin Cvejic, Jesse Jansen, Tessa Copp

**Affiliations:** 1 School of Psychology, Faculty of Science, The University of Sydney, Sydney, NSW 2006, Australia; 2 Sydney Health Literacy Lab, School of Public Health, Faculty of Medicine and Health, The University of Sydney, Sydney, NSW 2006, Australia; 3 Department of Family Medicine, School Care and Public Health Research Institute (CAPHRI), Faculty of Health Medicine and Life Sciences (FHML), Maastricht University, 6229 ER Maastricht, The Netherlands

**Keywords:** polycystic ovary syndrome, common-sense model of self-regulation, illness perceptions, contraceptive use, physical activity

## Abstract

This paper aims to delineate the cognitive, emotional, and behavioural responses of women with polycystic ovary syndrome (PCOS) to their illness by applying the Common-Sense Model of Self-Regulation (CSM) to their health behaviour. An online cross-sectional design was used to examine the relationship between participants’ illness perceptions (illness identity, consequence, timeline, control, and cause) and emotional representations of their PCOS, and their health behaviours (diet, physical activity, and risky contraceptive behaviour). The participants were 252 women between the ages of 18 and 45 years, living in Australia, and self-reporting a diagnosis of PCOS, recruited through social media. Participants completed an online questionnaire regarding illness perceptions as well as their diet, physical activity, and risky contraceptive behaviour. Illness identity was positively associated with the number of maladaptive dietary practices (*B* = 0.71, 95% CI: 0.003, 0.138; *p* = 0.04), and perception of longer illness duration was associated with reduced physical activity (OR = 0.898, 95% CI: 0.807, 0.999; *p* = 0.49) and risky contraceptive behaviour (OR = 0.856, 95% CI: 0.736, 0.997; *p* = 0.045). The limitations of the study include all data being self-reported (including PCOS diagnosis), and the potential for analyses of physical activity and risky contraceptive use being underpowered due to reduced sample sizes. The sample was also highly educated and restricted to those who use social media. These findings suggest that illness perceptions may play a role in influencing health behaviour in women with PCOS. A better understanding of the illness perceptions of women with PCOS is needed to increase health-promoting behaviour and improve health outcomes for women with PCOS.

## 1. Introduction

Polycystic ovary syndrome (PCOS) is an endocrine disorder that affects 6% to 15% of reproductive-age women, depending on the diagnostic criteria used and the population studied [1]. It is associated with adverse reproductive, metabolic, and psychological features, such as decreased fertility, insulin resistance, type II diabetes mellitus, and depression and anxiety. Women with PCOS are more likely to be overweight, with one study finding that approximately 70% of women with PCOS were either overweight or obese [2]. The relationship between PCOS and weight is unclear, though some evidence suggests that obesity may play a causal role in inducing PCOS symptoms [3]. Further, weight loss of as little as 5% is associated with improvement in symptoms [4]. As a result, weight management (weight loss for women who are overweight, and weight maintenance for women who are at a healthy weight) is recommended as first-line therapy for PCOS through healthy lifestyle changes to diet and physical activity [5]. In addition, weight management supports the prevention of the development of adverse metabolic consequences [4]. However, there remain discrepancies in dietary and exercise behaviour among women with PCOS, with some studies finding that women with PCOS reported making adjustments to their diet and exercise but that it made little difference to their weight [6,7]. Other studies found that women with PCOS struggled to adhere to the dietary and physical activity recommendations given to them [8,9], and were more likely to have higher sedentary behaviour than women without PCOS [10]. Studies have also found that women with PCOS perceive greater susceptibility to weight gain [9], despite women with and without PCOS losing the same amount of weight after weight management interventions [11]. Additionally, Moran and colleagues found that women with PCOS were more likely to engage in maladaptive dietary practices, such as smoking, using laxatives or diuretics, and fasting [12]. They expressed concerns that alternative weight management practices may lead to the development of eating disorders in some women. More recent research has similarly found that women with PCOS are at higher risk of eating disorders [13,14].

PCOS is also associated with decreased fertility, which has been shown to be of concern to many women with PCOS [7,8], with many women reporting it to be the most distressing part of their PCOS diagnosis [15]. Although there is a greater need for fertility treatment, studies have found that women with PCOS have similar numbers of children as women without PCOS [16,17]. This suggests that women’s perceptions of infertility may be exaggerated, with Lin et al. finding that many women with PCOS believed they could not conceive [9]. Some research has also found that women with PCOS are less likely to use contraception than those without [8,17,18], potentially because they believe the likelihood of conceiving to be very low. However, reduced contraceptive use has not been observed in all studies [16].

While there is evidence that some women with PCOS may use maladaptive coping strategies, such as poor diet, low levels of physical activity, and risky contraceptive behaviour [8,17], the reasons for this have not been thoroughly investigated and are not well understood. The Common-Sense Model of Self-Regulation (CSM) is a theoretical framework that suggests that when an individual is presented with a threat to their health, they form common-sense beliefs about their illness [19]. These illness perceptions or representations are then used by the individual, alongside their emotional responses to their illness, for coping and managing their illness.

Illness perceptions can be broken down into five categories: identity, cause, consequence, timeline, and control [20]. Identity refers to the illness label and associated symptoms. Cause is the individual’s perceptions of what caused their illness. Timeline is how long the individual believes their illness will last. Consequence refers to the effects the individual anticipates their illness will have on their life. Control refers to whether or not the individual believes their illness can be cured or controlled by themselves or medical professionals. These illness perceptions function in parallel with an individual’s emotional response to influence their behaviour.

Studies have shown that an individual’s illness perceptions and emotional responses are useful for predicting their adherence to treatment, changes in lifestyle, and use of coping strategies [20,21,22]. Stronger illness identity and more severe emotional representations have consistently been associated with poorer coping strategies and adherence to treatment, while greater perceived control has consistently been linked with better coping strategies and adherence to treatment [21,22,23]. However, there is conflicting evidence with regard to the direction of the effects of timeline and consequences, with some studies showing that more chronic perceptions of timeline and severe perceived consequences lead to better coping strategies and treatment adherence [20,23,24,25,26], and others showing that they lead to poorer outcomes [22].

This study aimed to examine how illness perceptions and emotional representations are associated with coping behaviour in women with PCOS. Specifically, how perceptions of illness identity, consequence, timeline, control, cause, and distress relate to positive or negative behaviours with respect to diet, physical activity, and risky contraceptive behaviour.

## 2. Materials and Methods

### 2.1. Ethics Approval

The study received ethics approval from the University of Sydney Human Research Ethics Committee (2018/358).

### 2.2. Participants

The participants were 252 women between the ages of 18 and 45, living in Australia, and self-reporting a diagnosis of PCOS. Participants were recruited through social media (Facebook advertising). An a priori power analysis suggested that a sample of this size would be sufficient to provide approximately 80% statistical power to detect small effect sizes (f2 = 0.059) in multiple regression modelling, with an alpha of 0.05.

### 2.3. Design

Using an online cross-sectional design, this study examined how participants’ illness perceptions and emotional representations of PCOS are associated with their diet quality, physical activity, and risky contraceptive use.

### 2.4. Procedure

The study was conducted online using Qualtrics survey software. After providing informed consent, participants completed a questionnaire assessing PCOS-related health questions, illness perceptions and emotional representations, behavioural outcomes and beliefs, followed by demographic and health questions. The study was piloted with a convenience sample of 24 women between the ages of 18 and 45, including women with PCOS, to test suitability. These responses were excluded from the analysis.

### 2.5. Measures

#### 2.5.1. Demographic and PCOS-Related Characteristics

Participants provided demographic details (age, gender, whether living in Australia, education level, BMI (calculated from height and weight), if they had ever given birth, were currently trying to conceive, currently pregnant, and had ever been diagnosed with a chronic physical or major mental illness), and background information regarding their PCOS diagnosis (age at diagnosis, symptoms present at diagnosis, current symptoms, treatment for PCOS, self-rated severity of PCOS, and the impact of PCOS on their life)

#### 2.5.2. Psychological and Cognitive Variables

##### Illness Perceptions and Emotional Response

A version of the previously validated Revised Illness Perception Questionnaire (IPQ-R) [27], adapted to PCOS, was used to measure each of the five illness perceptions and the emotional response. The illness perception subscales displayed moderate (α = 0.78; 0.70; identity and control, respectively) to high (α = 0.82; 0.83; 0.89; timeline, consequence, and emotional response, respectively) internal reliability in the current study.

##### Psychological Distress

Psychological distress was measured using the validated Kessler Psychological Distress Scale (K-10) [28] based on 10 items, measured on a 5-point scale: 1 = *none of the time* to 5 = *all of the time*, summed (range: 10–50). The K-10 showed high internal reliability (α = 0.93).

##### Beliefs about Fertility

Two questions assessed beliefs about fertility. The first assessed the perceived importance of using contraception (single item: *“It is important for me to use contraception to prevent pregnancy”*) and the second assessed beliefs about the ability to become pregnant naturally (single item: *“It is impossible for me to fall pregnant naturally”*) each on a 5-point scale (1 = *strongly disagree* to 5 = *strongly agree*).

#### 2.5.3. Behavioural Outcome Variables

##### Diet Quality

Diet quality was measured using the 13-item Diet Quality Tool (DQT), which has been previously validated in cardiovascular disease [29]. Composite scores were categorised into high, moderate, and low compliance with dietary guidelines.

##### Maladaptive Diet

Maladaptive diet was measured using a single-item scale from the Australian Longitudinal Survey on Women’s Health (ALSWH) [30]. The scale assessed 11 methods of maladaptive weight control (e.g., *vomited on purpose after eating* and including *none of the above*). Maladaptive diet scores were summed (excluding *none of the above*), with higher scores indicating more maladaptive diets.

##### Physical Activity

Physical activity was measured using the previously validated International Physical Activity Questionnaire Short Form (IPAQ-SF) [31]. The IPAQ-SF consisted of 7 items, which assessed how much time participants spent doing either vigorous physical activity, moderate physical activity, walking, or sitting, during one week. All times (excluding sitting) were converted into minutes, weighted, and summed. Scores were then categorised into high, moderate, or low levels of physical activity [31].

##### Risky Contraceptive Behaviour

Risky contraceptive behaviour was measured using one item from the ALSWH 1989–95 cohort [32]. After first assessing whether participants had ever had sex, participants who reported ‘yes’ were asked what type of contraception they used, listing 13 contraception types, which were categorised into safe, risky (*emergency contraception, withdrawal method, safe period method,* and *other*), and none (*I don’t use contraception*).

### 2.6. Data Analyses

All analyses were conducted using Statistical Packages for Social Sciences (SPSS) Version 22.0. Adjacent categories with low response frequencies were collapsed where appropriate. A series of multivariable regression models were used to test the hypotheses, with *p*-values less than 0.05 considered statistically significant. Age, education, and BMI were controlled for in all models. Multivariable linear regression was used to test the effect of illness perceptions and emotional representations on participants’ resort to maladaptive dietary practices. Binary logistic regression was used to test the effect of illness perceptions and emotional representation on participants’ diet quality (DQT). Two ordinal logistic regressions were conducted to test the effect of illness perceptions and emotional representations on participants’ physical activity (IPAQ-SF) and the use of risky methods of contraception, respectively.

## 3. Results

### 3.1. Demographics and PCOS-Related Characteristics

Of the 258 participants who responded to the survey, six participants were excluded due to incomplete responses (*n* = 1) or failing to meet eligibility criteria (*n* = 5), resulting in a final sample size of 252. The demographic characteristics are shown in Table 1. The sample ranged in age between 18 and 45 years (M = 27.6, SD = 6.4). BMI ranged from 17.9 to 57.8 (M = 31.2, SD = 8.7). The majority of the sample had either overweight (20.9%, *n* = 54) or obese BMIs (46.1%, *n* = 119). The mean psychological distress score was 25.6 (SD = 9.5), indicating moderate psychological distress (scores ranging from 25 to 29 indicate a moderate mental disorder; Table 2).

### 3.2. Illness Perceptions and Behavioural Outcomes

The illness perceptions and behavioural outcome variables are displayed in Table 2.

### 3.3. Association between Explanatory Variables

Table 3 shows the correlations between the continuous explanatory variables. All of the illness perceptions were moderately correlated with each other and with emotional response, except for timeline and identity, and control and consequence, both of which pairs were weakly correlated. BMI was weakly correlated with all other explanatory variables except control, which was not statistically significant. Age was weakly correlated with consequence, control, and BMI.

### 3.4. Factors Associated with Behavioural Outcomes

#### 3.4.1. Diet Quality

The results from multivariable regression modelling for all outcomes are shown in Table 4. BMI was the only explanatory variable associated with poor diet quality. For every one-unit increase in BMI, the odds of having low compliance with dietary guidelines increased by 4.9% (95% CI: 1.3 to 8.6%; *p* = 0.007) compared to moderate to high compliance. After controlling for age, BMI, and education, neither any of the illness perceptions (identity, consequence, timeline, and control) nor emotional representation were significantly associated with a healthy diet.

#### 3.4.2. Maladaptive Diet

After controlling for age, education and BMI, illness identity was significantly associated with the number of maladaptive dietary practices. With each one-unit increase in illness identity (with higher identity scores indicating that participants perceived more of their symptoms to be related to their PCOS), the number of maladaptive dietary practices increased on average by 0.071 (95% CI: 0.003 to 0.138; *p* = 0.04). BMI was also a significant correlate of the number of maladaptive dietary practices. The number of maladaptive dietary practices increased on average by 0.039 (95% CI: 0.012, 0.065; *p* = 0.004) per kg/m^2^ increase in BMI. No other variables were independently associated with the number of maladaptive dietary practices.

#### 3.4.3. Physical Activity

Timeline was significantly associated with physical activity after controlling for age, education, and BMI, with a more chronic perception of PCOS (reflected by higher timeline scores) associated with reduced odds of higher physical activity levels (OR = 0.898, 95% CI: 0.807, 0.999; *p* = 0.049). No other variables were associated with physical activity.

#### 3.4.4. Risky Contraceptive Behaviour

Timeline was associated with reduced risky contraception behaviour. For each one-unit increase in the perceived timeline (with higher timeline scores indicating participants perceive their PCOS to be more chronic), the odds of using a more risky method of contraception were lower (OR = 0.856, 95% CI: 0.736, 0.997; *p* = 0.045). By contrast, older age was associated with more risky contraceptive behaviour. For every year increase in age, the odds of using a riskier method of contraception increased by a factor of 1.104 (95% CI: 1.035, 1.177; *p* = 0.003). No other variables were associated with differences in risky contraceptive use.

## 4. Discussion

This study found that illness perceptions from the Common-Sense Model of Self-Regulation were associated with maladaptive diet, physical activity, and risky contraceptive behaviour in women with PCOS, with stronger illness identity (participants believing that a greater number of symptoms were related to their PCOS) being associated with maladaptive dietary practices, and timeline being associated with engagement in physical activity and risky contraceptive behaviour. Neither any of the illness perceptions nor emotional representation were associated with diet quality. BMI, however, was a significant correlate of diet quality, with increases in BMI associated with decreases in diet quality.

Stronger illness identity was associated with a higher number of maladaptive dietary practices. This supports findings that stronger illness identity leads to poorer coping strategies [21] and that women with PCOS, particularly those with more symptoms, may be at an increased risk of eating disorders [13,14]. BMI was also associated with maladaptive diet, with higher BMI associated with greater numbers of maladaptive dietary practices. Taken together with previous research findings, these findings suggest that some women with PCOS may adopt more maladaptive dietary practices and struggle to adhere to dietary recommendations [8,9]. Clinicians should look out for signs of maladaptive dietary practices in women with PCOS, particularly those with more symptoms or who have a high BMI [34].

We found that timeline was associated with engagement in physical activity, so that for participants who perceived the timeline of their PCOS to be less chronic and more acute, engagement in physical activity was increased. This supports Hagger and Orbell’s study, which found that illness timelines that were perceived as more chronic led to poorer coping strategies [22]. Implementing healthy behaviour changes and maintaining changes over time is a serious challenge [35], with rates of nonadherence to treatment plans for chronic illness reported to be as high as 50–80% [36,37]. More multifaceted approaches to promoting long-term adherence are needed: for instance, interventions with several elements, such as increased social support, addressing health literacy, skills training, person-centred goals, and addressing individual barriers, including mental health [36,37,38].

By contrast, timelines that were perceived to be more chronic were associated with the use of less risky methods of contraception. This contrast may be explained by the smaller sample for the risky contraception analysis (*n* = 158), due to removing participants who reported not having had sex, or who were currently trying to conceive or pregnant, meaning these results should be interpreted with caution. Age was found to be associated with risky contraception methods; as age increases so does the use of more risky methods of contraception. This supports other studies which have found that women who do not use contraception are more likely to be aged 40 years or over, potentially because they believe they have a lower risk of getting pregnant or perhaps the anticipated consequences of unintended pregnancy are less severe [39,40,41].

Of the participants in this study, 45.2% reported that they believed genetics/hereditary factors to be the number one cause of their PCOS, which was the most commonly reported perceived cause of PCOS. Whilst genetic susceptibility likely plays an important role [42], women who believe their PCOS is solely determined by their genes may not believe that changes to their diet and physical activity levels will make a difference [43], despite evidence showing that weight loss is associated with improvement in PCOS symptoms [4]. Indeed, Lin et al. found that women with PCOS were less likely to believe that physical activity and a healthy diet can prevent negative health outcomes, such as weight gain, than women without PCOS [9].

Interestingly, illness perceptions of consequences and control, and emotional representation were not associated with any of the health behaviour outcomes. This conflicts with findings that greater perceived control leads to better coping strategies and more severe emotional representations lead to poorer coping strategies [21]. In this study, emotional representation was moderately correlated with all illness perceptions, while control and consequence were moderately correlated with illness identity. This level of intercorrelation may have impacted the ability to detect statistically significant associations with the outcomes, as the individual illness perceptions uniquely explain only a small amount of the variability in the outcome. A recent study that looked at the relationship between illness perceptions and psychological distress in women with PCOS found that stronger illness identity and greater perceived consequences were associated with greater psychological distress and greater perceived control was associated with lower psychological distress [44]. This is consistent with the correlations found between emotional response and the illness perceptions of identity, control, and consequence in the current study.

Although we did not look at associations between illness perceptions and psychological distress in the current study, participants reported relatively high emotional responses to their PCOS and had moderate psychological distress scores on the K10, despite 77.5% describing the severity of their PCOS as mild or moderate, and 57.7% reporting that their PCOS had a minimal or moderate impact on their life. This is consistent with the findings of Hahn et al. that psychological distress was not strongly associated with any particular features of PCOS, suggesting any woman with PCOS could be susceptible to increased distress regardless of the type or severity of symptoms [45].

### 4.1. Strengths and Limitations

The current study provides a novel approach to understanding the illness perceptions and health behaviours of women with a broad spectrum of PCOS symptoms. To the authors’ knowledge, this is the first study to apply the Common-Sense Model of Self-regulation and the Illness Perception Questionnaire, in order to better understand how women’s perceptions of PCOS influence their diet, physical activity, and contraceptive use. A strength of the study was the use of Facebook to recruit participants from the community across the spectrum of symptom severity. Most of the previous literature on PCOS has recruited women from specialist clinics, leading to an over-representation of women with more severe symptoms of PCOS [46,47], reducing generalisability. Through social media advertising, this study was able to recruit women reporting a wide range of PCOS symptoms, severity, and impact on life, with 29.8% of our sample describing their PCOS as mild, 47.7% as moderate, and 17.8% as severe/debilitating.

There are some limitations to the current study. All the data are self-reported, including PCOS diagnoses. The sample was also highly educated, with 58.5% of participants having completed a Bachelor’s degree or higher, compared to 32.0% of Australians in the general population [48]. Additionally, 69.2% of the sample were overweight or obese. Although this aligns with the general Australian population [49] and estimates in PCOS populations [2], it may limit the generalisability of findings to women with a lower BMI. Furthermore, participants were limited to an age range of 18–45 and recruited through Facebook, excluding teenagers, older women who previously had PCOS during their reproductive years, and those who do not use social media. Further, although we examined emotional representations, general psychological distress was not included as an explanatory variable in the statistical models. Studies in both hospital and community samples have found many adverse psychological features associated with PCOS [13,50,51,52,53], which may hamper women’s ability to undertake and maintain healthy behaviour.

Another limitation is that there were 51 missing answers for physical activity due to participants answering “unsure” for times spent on one or more types of physical activity, leaving a total sample size of 201 out of 252 participants for the analysis of physical activity. Along with the smaller sample for the risky contraception analysis, this may have left these analyses underpowered.

### 4.2. Implications

These findings may help clinicians identify women at higher risk of poorer coping strategies and more maladaptive health behaviour. In addition, awareness of the impact of these illness perceptions on self-management behaviour may help inform future, more holistic PCOS interventions aiming to improve self-management and health outcomes. For example, improved information provision, health literacy, and education about PCOS may allow women to form illness perceptions associated with more positive management outcomes. Furthermore, women need education and multidisciplinary support to help them maintain a healthy lifestyle long-term, including access to professional and social support in overcoming barriers to a healthy lifestyle, such as depression and anxiety.

## 5. Conclusions

This study found that a stronger illness identity and timeline (i.e., the perception of PCOS as more chronic) were associated with a more maladaptive diet, and physical activity, whilst a more acute perceived timeline was associated with more risky contraception use. This suggests that illness perceptions may play a role in influencing health behaviour in women with PCOS. A more thorough understanding of how and why these illness perceptions were associated with health behaviour outcomes is needed, in order to create useful interventions for women with PCOS. More specifically, to help improve the diet quality and physical activity levels of women with PCOS as an important part of their treatment, and to reduce the rates of risky contraception use so as to prevent unplanned pregnancy.

## Figures and Tables

**Table 1:** Demographic and self-reported PCOS-related characteristics of participants (*n* = 252). Data are displayed as *n* (%) unless otherwise specified.

	M	SD
**Age** (18–46 years)	27.6	6.4
	***n***	**%**
**Education**		
Below Year 12	6	2.4
Year 12 or equivalent	49	19.4
Certificate/Diploma	46	18.3
Bachelor’s degree or higher	151	59.9
**Relationship**		
Single/Never married	71	28.2
In relationship	175	69.4
Previously married	6	2.4
**Given birth**		
Yes	45	17.9
No	207	82.1
**Trying to conceive**		
Yes	49	19.4
No	203	80.6
**Currently pregnant**		
Yes	6	2.4
No	246	97.6
**BMI**		
Underweight	4	1.6
Healthy weight	73	29.2
Overweight	54	21.6
Obese	119	47.6
**Perceived severity of PCOS**		
Unnoticeable	6	2.3
Mild	77	29.8
Moderate	123	47.7
Severe/Debilitating *	46	17.8
**Impact of PCOS on life**		
No impact/Minimal *	46	17.8
Moderate	103	39.9
Significant	75	29.1
Extremely Significant	28	10.9
**Current symptoms** ^†^		
Polycystic ovaries on ultrasound	165	64.0
Weight gain	160	62.0
Irregular or absent menstrual cycles	158	61.2
Excess male pattern hair growth	125	48.4
Acne	89	34.5
Difficulty becoming pregnant	68	26.4
Hair loss or male pattern balding	24	9.3
Other	33	12.8
**Treatment** ^†^		
Diet and exercise	198	76.7
Oral contraceptive pill	166	64.3
Diabetes medication	88	34.1
Fertility medication	42	16.3
Herbal supplements	42	16.3
Anti-testosterone medication	19	7.4
Other	32	12.4
**Perceived causes of PCOS**		
Genetic/hereditary factors	114	45.2
Weight/weight gain	19	7.5
Hormones	16	6.3
Diet	12	4.8
Medication	7	2.8

* Categories collapsed due to low response frequencies, ^†^ Multiple response options possible for these items.

**Table 2:** Psychological and cognitive variables, and behavioural outcome variables of the sample (*n* = 252). Scale ranges are indicated in parentheses.

	M	SD
**Psychological and cognitive variables**		
**Psychological distress (K10)** (10–48) *	25.6	9.5
**Illness Perceptions (IPQ-R)**		
Illness identity (0–19)	8.7	3.8
Consequence (6–25)	17.6	3.9
Timeline (13–25)	20.8	2.9
Control (4–20)	11.5	2.8
Emotional Response (7–30)	20.5	5.3
	***n***	**%**
**Beliefs about fertility**		
**Important to use contraception** ^†^		
Strongly disagree/Disagree ^§^	17	10.2
Neither agree nor disagree	24	14.4
Agree	49	29.3
Strongly agree	71	42.5
**Impossible to become pregnant naturally** ^†^		
Strongly disagree	11	6.3
Disagree	51	29.3
Neither agree nor disagree	76	43.7
Agree/Strongly agree ^§^	30	17.2
	**M**	**SD**
**Behavioural outcome variables**		
**Maladaptive dietary practices** (0–8)	3.0	1.7
	***n***	**%**
**Diet Quality (DQT)**		
Low	150	58.1
Moderate/High ^‡^	102	39.5
**Physical Activity (IPAQ-SF)** ^§^		
Low	46	17.8
Moderate	62	24.0
High	93	36.0
**Risky Contraceptive Behaviour** ¶		
Safe	112	43.4
Risky	20	7.8
None (no contraception used)	26	10.1

* According to the National Survey of Mental Health and Wellbeing, the population mean for women is 15.0 and SE is 0.1 [33], ^†^ Excluding participants who reported difficulty becoming pregnant currently, when diagnosed, or when first seeking medical care, and participants currently trying to conceive or pregnant, ^‡^ Categories collapsed due to low response frequencies, ^§^ Excluding participants who reported that they were unsure of how much time they spent on at least one type of physical activity, ¶ Excluding participants currently trying to conceive or pregnant, and participants who reported that they had never had vaginal sex.

**Table 3:** Correlation matrix of explanatory variables (*n* = 252).

	Identity	Consequence	Timeline	Control	Emotional Response	BMI	Age
Identity	1.000						
Consequence	0.478 *	1.000					
Timeline	0.209 *	0.377 *	1.000				
Control	−0.307 *	−0.248 *	−0.470 *	1.000			
Emotional response	0.436 *	0.565 *	0.301 *	−0.301 *	1.000		
BMI ^†^	0.240 *	0.281 *	0.181 *	−0.109	0.166 *	1.000	
Age	0.081	0.205 *	0.074	−0.163 *	−0.081	0.174 *	1.000

* Correlation is significant at the 0.01 level, ^†^ BMI (*n* = 250).

**Table 4:** Regression-based estimates for all outcomes.

Explanatory Variable	Outcome			
	Diet Quality OR (95% CI)	Maladaptive Diet Mean Difference (95% CI)	Physical Activity ^†,‡^ OR (95% CI)	Risky Contraceptive Use ^†,§^ OR (95% CI)
**Illness Perceptions**				
Identity	1.04 (0.96, 1.13)	0.07 * (0.00, 0.14)	0.97 (0.90, 1.06)	1.01 (0.90, 1.13)
Consequence	1.01 (0.92, 1.11)	0.03 (−0.04, 0.11)	0.98 (0.89, 1.08)	1.00 (0.87, 1.14)
Timeline	1.04 (0.93, 1.16)	0.05 (−0.04, 0.13)	0.90 * (0.81, 1.00)	0.86 * (0.74, 1.00)
Control	1.04 (0.93, 1.16)	−0.02 (−0.11, 0.07)	0.98 (0.87, 1.10)	0.91 (0.76, 1.08)
Emotional response	1.00 (0.94, 1.07)	−0.01 (−0.06, 0.04)	1.04 (0.97, 1.11)	0.97 (0.88, 1.06)
**Control variables**				
Age (year)	1.01 (0.96, 1.06)	−0.03 (−0.07, 0.01)	0.98 (0.93, 1.02)	1.10 ** (1.06, 1.18)
Body mass index (kg/m^2^)	1.05 ** (1.01, 1.09)	0.04 ** (0.01, 0.07)	0.97 (0.94, 1.01)	1.02 (0.98, 1.07)
**Education**				
High school or less	1.00 (reference)	-	-	-
Certificate/Diploma	1.12 (0.45, 2.81)	0.48 (−0.23, 1.18)	1.09 (0.43, 2.77)	0.47 (0.13, 1.64)
University	0.71 (0.34, 1.45)	0.48 (−0.11, 1.06)	1.36 (0.65, 2.85)	0.48 (0.17, 1.37)

^†^ Ordinal logistic regression models, * *p* ≤ 0.05, and ** *p* < 0.01, ^‡^ Participants who responded “unsure” to at least one IPAQ-SF item were excluded from this analysis; *n* = 201, ^§^ Excludes participants currently trying to conceive or pregnant, and participants who reported that they had never had vaginal sex; *n* = 158.

## Data Availability

Data are available upon reasonable request.
